# Flatwise to Upright Build Orientations under Three-Point Bending Test of Nylon 12 (PA12) Additively Manufactured by SLS

**DOI:** 10.3390/polym14051026

**Published:** 2022-03-03

**Authors:** Marius Nicolae Baba

**Affiliations:** Department of Mechanical Engineering, Faculty of Mechanical Engineering, Transilvania University of Brașov, 500036 Brasov, Romania; mariusbaba@unitbv.ro

**Keywords:** Nylon 12 (PA12), additive manufacturing (AM), selective laser sintering (SLS), three-point bending, bending strength, flexural elasticity modulus, characteristic stress, Weibull modulus

## Abstract

This paper describes in detail a series of static tests conducted in a three-point bend configuration on three build orientations (i.e., 0°, 45°, and 90°) of additively manufactured Nylon 12 (PA12) specimens produced with a powder refresh ratio of 50%, using a benchtop industrial SLS platform, Formlabs Fuse 1. The bending strength and flexural elasticity moduli are determined following ISO 173 specifications and by employing a more complex equation that considers the influence of large deflections as per ISO 14125 indications. Statistical variability of experimental data is considered and compared to the results from the literature. Through a fractographic SEM study, the damage morphologies of tested specimens are analyzed and associated with the recorded load-deflection curves for an accurate perception of build orientation-dependent anisotropy in bending properties of AM PA12 SLS specimens. A surprising result of this investigation is that the specimens built with 45° orientation showed superior modulus elasticity in flexure but a low bending strength compared to flatwise oriented specimens. In addition, a Weibull reliability quantification of bending strength is adapted to pinpoint the effects of internal 3D printing flaws (contained within a characteristic highly-stressed volume of material) over the failure probability of the three build orientations in question.

## 1. Introduction

With the emergence of additive manufacturing technologies alongside the integration of CAD/CAE software systems, the trend of 3D printing is nowadays rapidly expanding from artisanal fabrication toward large-scale production of high-performance functional components intended to replace the conventionally manufactured parts for various and different demanding applications. Additive manufacturing (AM) virtually involves building up layer upon layer of material, making it possible to create much lighter but stable structural components with complex geometries that are excessively difficult or even impossible to be processed through the use of conventional subtractive fabrication methods. Besides, the actual AM techniques and their specific features ensure sustainable conservation of the environment and its resources by an effective recycling system that keeps materials in use for as long as possible. The main features of distinct AM techniques, different AM plastic materials, and their applications, along with the importance of studying the effects of process parameters on the characteristics of 3D printed parts, are presented in detail by Lee et al. [1], Mousavi Nejad et al. [2], Vanaei et al. [3], Schmidt et al. [4], Starr et al. [5], and Wu et al. [6].

Within the whole range of technologies based on the principles of additive manufacturing, the selective laser sintering (SLS) is among the most versatile approaches that typically use laser energy as a heat source to locally melt and fuse the polymer powder particles, such as, but not only, nylon or polyether ketone [7]. Known also as laser beam powder bed fusion (LB-PBF), it has the ability to produce relatively large parts with good mechanical properties [8] and higher dimensional accuracy [9], especially compared with nozzle-based polymer deposition systems [10]. Moreover, the advantage of freedom design in components and production, afforded by the nature of the SLS process, enables the effective handling of internally nested shapes without extra supporting structures and the possibility to print multiple parts in the same volume [11]. In this respect, it is worth mentioning that Polyamide 12, also called by the acronym PA12, is an environmentally friendly polymer widely used for sintering applications due to its easy melting using lasers, compared with other polymers [12,13,14,15,16,17,18]. In its bulk form (i.e., powder), it can be found under different commercial names, from which the most popular are PA 2200 (from EOS, Krailling, Germany), Duraform PA (from 3D-Systems, Rock Hill, SC, USA), or Nylon 12 (from Formlabs, Somerville, MA, USA).

Data reported in the literature reveals that the mechanical properties of PA12 manufactured by SLS may vary broadly depending on the process parameters and the anisotropy, the latter being directly linked to the particular direction about which the 3D build orientation was carried out. As recently highlighted well in the paper [19] published by Lammens et al., the PA12 SLS material response to tensile loading and the specific issues related to acquiring qualitative and reproducible results are critically analyzed, emphasizing the effects of material anisotropy. The specimens were manufactured with an EOS P 395 printing platform from PA2200 powder using a mixture recycled/virgin ratio of 50/50 and an alternate x–y scanning pattern. Based on digital image correlation and post-processing of standard tensile tests, accurate strain measurements within the elastic-plastic range up to the final failure were obtained. Their results proved a significant viscous contribution in the isotropic elastic response with orthotropic failure properties.

Earlier studies in PA12 sintering have tried to analyze and compare the directional mechanical properties of test specimens [20,21,22,23]. The pioneering work of Ajoku et al. [22] is particularly relevant to the topic under discussion here since it addresses the effects of building orientation on the mechanical properties of AM PA12 SLS under tensile, compression, and three-point bending tests. The specimens manufactured using virgin powder were produced with a laser-sintering machine (3D Systems Vanguard SI), about three distinct orthogonal build orientations about x-, y- and z-axes relative to a fixed x-printing direction were tested to assess their directional strength and stiffness response under static loading conditions. By looking specifically at their bending response results, a maximum difference of 9.4% in the ultimate strength and 7% in the flexural elasticity modulus was reported. However, neither characteristic stress-strain curves nor load-deflection plots intended to characterize graphically the directionally dependent nonlinear material response were provided. All the same, they described in detail the so-called end-vector-effect, which typically occurs due to different degrees of laser beam exposure between the start and the end of each sintered scan layer. Essentially, according to authors, it occurs for thin sections whose dimensions along printing direction are small, as the short bursts of energy experienced at the turning points lead to sintered parts that are much more dense at the boundary surfaces than to the core regions. Caulfield et al. [23] studied the effects of energy density level (which combines the influences of laser power magnitude with the hatch spacing and beam speed) within the SLS process on the mechanical response of polyamides manufactured using a DTM Sinterstation 2500^plus^ with DuraForm TM Polyamide powder. No information about the powder mixing ratio (recycled to virgin) was specified. They concluded that the specimens manufactured using high energy density levels exhibit a more ductile behavior than those obtained at low energy densities. Variations of mechanical properties and the specific failure modes of AM specimens built along 0° and 90° orientations (i.e., x- and z-axes) were also addressed in their work, emphasizing the results of standard tensile test data. Greater elasticity modulus and tensile strength values were reported along the primary x-axis than the secondary z-axis. In addition, the yield strength of specimens of 90° orientation exhibit a pronounced dependence on the energy level than the specimens with 0° orientation. For the broad range of energy density tests, it was concluded that the specimens built with 0° orientation had less elongation at fracture, and higher ultimate tensile strength relative to specimens with 90° build orientation.

More recently, Hofland et al. [24] used the PA 2200 powder with a recycled/virgin mixture ratio of 50/50 to manufacture specimens with 0° and 90° build orientations. Based on their standard tensile tests carried out according to ISO-527-2, they demonstrated that the directional dependence of mechanical properties becomes more pronounced with the increase of energy input. Phillips et al. [25] have performed three-point bending tests on specimens made of three different additive manufacturing techniques: FDM, SLS, and Polyjet. The SLS specimens were produced in a ProX 500 SLS machine using Duraform ProX PA12 powder with a recycled/virgin mixture ratio of 15/85. Printing direction was alternated layer by layer between the horizontal x and y-axes. Their results indicate that each 3D printing technique produces distinct and asymmetric responses when comparing tension to compression loading regimes. Based on the obtained stress-strain curves, they calibrated some specific material models intended to be used in numerical simulations. However, they did not report any statistical data for bending strength and flexural modulus values.

As briefly reported above, although many works have been completed to date to investigate the mechanical properties of additively manufactured PA12 SLS materials, especially under tensile and/or compression tests, apparently little interest has been given to their anisotropic flexural properties [26]. Appendix A (see Table A1) summarizes the most representative flexural properties reported so far in the scientific literature [22,27,28,29,30,31,32]. It includes relevant information about the SLS platform and powder type, print orientation, laser power magnitude, layer thickness, and not the last, the employed testing standard and specimen dimensions. One should note that the derived AM composites based on PA12 SLS are not included in the table as they are beyond the scope of this study.

Since the directionally-dependent flexural properties are impetuously needed during the structural design phases, the lack of available material data can create technical issues when employing the up-and-coming AM technologies to produce part geometries subjected predominantly to simple bending. In such a context, it is worth mentioning that when a specimen made of longitudinally printed PA12 undergoes bending, failure is not likely to occur within the small deflection range. Thus, the elementary bending theory of beams becomes inadequate due to finite deformations and changes in the directions of reaction forces at the supporting rollers. As far as the authors’ knowledge, neither further references have been found linking the influence of large deflections to the response of PA12 specimens under three-point bending tests, nor empirical research was undertaken on the mechanical behavior of Polyamide material when processed on a benchtop industrial SLS platform (Formlabs Fuse 1) using a Nylon 12 sintering powder.

To address the literature research limitations mentioned above, in this paper, a comprehensive experimental investigation of static bending properties of PA12 specimens made of Nylon 12 sintering powder, and processed with a Formlabs Fuse 1 SLS platform, is considered. On account of this, the effects of flatwise (i.e., 0°), upright (i.e., 90°), and 45° flat-to-upright build orientations on the bending response of PA12 produced by SLS, under the three-point bending test, are considered in line with the effects of large deflections on the experimentally measured bending properties. The main statistical parameters associated with the elasticity modulus in flexure and bending strength, the specific flexural failure modes, and the strength reliability for the build orientations in question will be analyzed.

## 2. Materials and Methods

Rectangular beam-type specimens were printed for three-point bending tests, using a benchtop industrial SLS platform (Formlabs Fuse 1), with a Nylon 12 sintering powder produced by Formlabs Inc. Somerville, MA, USA. The Fuse 1 system employs a patent-pending technology termed Surface Armor which consists of a semi-sintered cover that preserves the area in the vicinity of the parts under processing being heated evenly. Thus, it ensures consistent mechanical properties, an excellent surface finish, and the possibility to use an effective powder refresh rate [33].

### 2.1. Specimen Production

**Figure 1:**
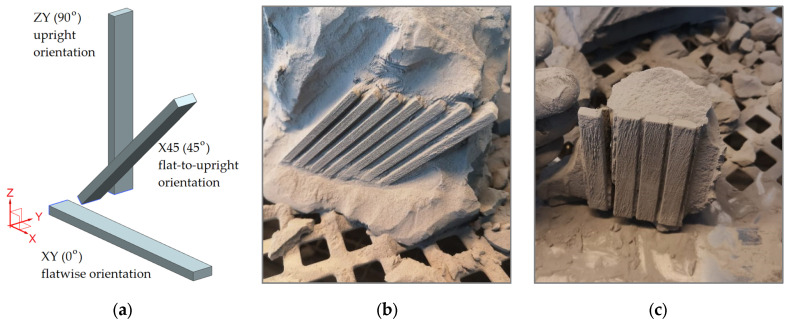
Designed and processed specimens: (**a**) CAD models defining the three build orientation; (**b**) X45 (flat-to-upright) processed specimens; (**c**) XZ (upright) processed specimens.

As shown in Figure 1a, three different build orientations were considered for this study, all of them being taken relative to the X-printing direction within the XY-plane. The first letter in the specimen orientation label refers to its longitudinal axis, while the second letter returns the width direction.

The specimens with on-edge orientations about Y-direction are excluded from this study to steer clear of the end-vector effects. The main printing parameters, such as the layer thickness (0.1 mm), the scanning speed (2000 mm/s), and the laser power magnitude (5 W), remained unchanged during the additively manufacturing procedure, while the internal temperature varied during processing between 178.3 °C and 178.6 °C. To keep away from possible manufacturing defects and the likelihood of print failures, a powder refresh ratio of 50% was considered, as Formlabs recommends an interval of 30% to 50% for the ratio of fresh to used powders [34,35].

The geometric dimensions of specimens with 10 mm in width, 80 mm in length, and 4 mm thickness were established as per ISO 173-2019 standard [35]. The CAD model of specimen geometry was prepared in Simcenter 3D and exported to Formlabs’ PreForm software as an STL file. Seven specimens were printed for each build orientation mentioned above to derive mean values and standard deviations for bending strength and flexural modulus.

Within the AM process of PA12 bending specimens, the Fuse 1 printer preheats the powder as it spreads up in a thin layer upon the top of the sintering platform in the building chamber towards a temperature level slightly below the raw material’s melting point. That way allows the laser source to raise the temperature over the specific areas of the powder bed more straightforwardly, whereas tracing the 3D model to solidify the parts. In essence, when scanning the geometry of the specimens, the laser heats the powder to the melting point of the involved raw material and consequently fuses the particles to form the solid parts. After printing, the build chamber was moderately cooled down, firstly inside the print enclosure and then outside the printer, to avoid warping phenomena and ensure the optimum mechanical properties. At the end of the printing process, the state of 45° flat-to-upright and 90° upright bending specimens just before removing the finished parts from the build chamber, are illustrated in Figure 1b,c, respectively.

Finally, the specimens were removed from the building chamber, separated, and cleaned from the excess powder with a compressed air station, followed by a sandblast post-processing carried out using glass balls of 100 to 200 μm.

### 2.2. Testing Procedure

**Figure 2:**
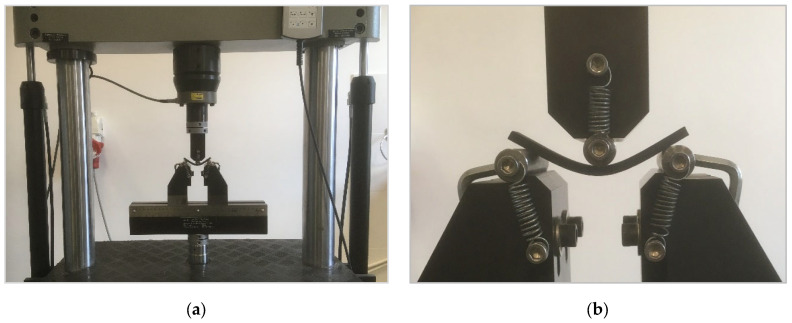
The three-point bending test set-up: (**a**) General view of test fixture assembly and loading frame; (**b**) Detailed view of the deflected specimen upon the supporting rollers.

The three-point bending tests were performed at room temperature using a dual column closed-loop Multipurpose Servohydraulic Universal Testing Machine, type LVF 50-HM (Walter–Bai A.G., Löhningen, Switzerland) equipped with a loading cell of 50 kN capacity (see Figure 2a).

The testing routine and the three-point fixture configuration followed strictly the specifications described by the ISO standard 178-2019 [36]. During the tests performed under displacement control, the applied force required to bend the AM specimen and the vertical deflection that it was undergoing due to the imposed constant rate of 1 mm/min at the mid-span of the specimen were recorded until fracture. As represented in Figure 2b, the specimen resting upon two cylindrical supports is deflected through the crosshead loading cylinder, acting at its mid-span. The distance between the supports was settled to 65 mm, equally divided into two loading spans of 32.5 mm each.

### 2.3. Data Processing

Bending stress was obtained primarily from the equation based on the elementary beam theory, as indicated by ISO 178-2019, which is represented as follows:

(1) $$\sigma_{\mathrm{eb}}=\frac{3\mathrm{FL}}{{2\mathrm{bh}}^{2}},$$

where $\sigma_{\mathrm{eb}}$ is the bending strength in Newtons per square millimeter (MPa), F is the applied load in Newtons (N), L is the distance between the cylindrical supports in millimeters (mm), b and h are the width and thickness of AM specimen in millimeters (mm), respectively.

For a nonlinear material response in simple bending, it is tenable to assume that all cross-sections initially plane and perpendicular to the longitudinal axis of the specimen will remain plane and perpendicular after deformation (i.e., Euler–Bernoulli’s hypothesis). Thus, by neglecting the warping effects due to shear deformations, it follows that the longitudinal strain shall be distributed linearly over the specimen section. The strains obtained from the displacement of loading point will be denoted in what follows with $\varepsilon_{\mathrm{eb}}$, as they were derived simply on the assumptions mentioned above, according to ISO 178-2019 [36]:

(2) $$\varepsilon_{\mathrm{eb}}=\frac{6\mathrm{hs}}{L^{2}},$$

where, besides the terms already involved in the Equation (1), s represents the mid-point deflection of the specimen in millimeters (mm).

In addition, to account for the possible effects of large deflections experienced by three-point flexure specimens made of PA12 SLS, the equation standardized in ISO 14125-1998 [37] was considered to determine the corrected bending stress:

(3) $$\sigma_{\mathrm{ld}}=\frac{3\mathrm{FL}}{{2\mathrm{bh}}^{2}}\left[1+6{\left(\frac{s}{L}\right)}^{2}-3\left(\frac{\mathrm{sh}}{L^{2}}\right)\right],$$

while for the calculation of corrected strains due to the effects of large deflections, the following equation provided in ISO 14125-1998 was employed:

(4) $$\varepsilon_{\mathrm{ld}}=\frac{h}{L}\left[6\left(\frac{s}{L}\right)\;-\;24.37{\left(\frac{s}{L}\right)}^{3}+62.17{\left(\frac{s}{L}\right)}^{5}\right].$$

The modulus of elasticity was evaluated between two strain levels, as given by [37]:

(5) $$E_{b}=\frac{\sigma_{0.25\%}-\sigma_{0.05\%}}{\varepsilon_{0.25\%}\;-\;\varepsilon_{0.05\%}}=\frac{\Delta \sigma }{\Delta \varepsilon },$$

representing the slope of the elastic deformation region in the stress-strain diagram, with Megapascals (MPa) units. Thus, in Equation (5), the strain values $\varepsilon_{0.05\%}=0.0005$ and $\varepsilon_{0.25\%}=0.0025$ were imposed, while the corresponding stresses $\sigma_{0.25\%}$ and $\sigma_{0.05\%}$ were found by indexing and matching from the stress-strain values. Alternatively, in terms of load and deflection, substituting either the expressions (1) and (2), or (3) and (4), into the Equation (5), it becomes:

(6) $$E_{b}=\frac{{3L}^{3}}{{12\mathrm{bh}}^{3}}\;\cdot \;\frac{F_{0.25\%}\;-\;F_{0.05\%}}{s_{0.25\%}\;-\;s_{0.05\%}}=\frac{{3L}^{3}}{{12\mathrm{bh}}^{3}}\;\cdot \;\frac{\Delta F}{\Delta s},$$

where the values of forces denoted by $F_{0.25\%}$ and $F_{0.05\%}$ together with the associated deflections $s_{0.25\%}$ and $s_{0.05\%}$ correspond to the imposed strains of 0.0025 and 0.0005.

Assuming that different build orientations influence the volume fraction of some inherent printing flaws within the AM-produced specimens with similar dimensions, conventional methods of reporting their strength are becoming pretty inadequate. Hence, to obtain accurate predictions on the strength of PA12 three-point bending specimens manufactured by selective laser sintering (SLS) with different build orientations, Weibull statistics will be used hereafter. Essentially, its applicability relies on the fact that each specimen having a particular build orientation will have a unique distribution of printing flaws and consequently a unique strength. In addition, a transition from ductile to quasi-brittle fracture is expected with the change in build orientation of specimens, from 0 to 90 degrees. However, by employing the Weibull formulation, one can only evaluate the probability that specimens of a given build orientation will fail, whereas statistics are needed to describe the strength limits of untested specimens.

The ultimate bending stresses of PA12 SLS for each printing orientation (and the same volume of material) were analyzed using the Weibull formulation, which can be expressed through a two-parameter function [38]:

(7) $$P_{f}(\sigma )=1\;-\;\exp\left[-\overset{}{\underset{V}{\int }}{\left(\frac{\sigma }{\sigma_{0}}\right)}^{m}\cdot \mathrm{dV}\right],$$

where $P_{f}\left(\sigma \right)$ denotes the probability of failure, σ is the applied stress at a given point in the volume of the specimen, $\sigma_{0}$ is the characteristic stress, representing the stress value at which the probability of failure equals 63.2%, while m is a constant known as the Weibull modulus. The above integral must be evaluated over the volume of specimen effectively stressed in tension due to the applied given loading compared to a pure uniaxial stress state. High values of Weibull modulus imply consistent strength data, that is, a narrow distribution of printing defects and thus a lower scatter in the data, whereas the small values indicate significant variations in strength and, therefore, a significant spread of data.

The data processing theoretical background for reliability assessment of bending strength is described in Appendix B, based on the references [39,40,41].

## 3. Results

### 3.1. Load-Deflections and Bending Stress-Strain Curves

**Figure 3:**
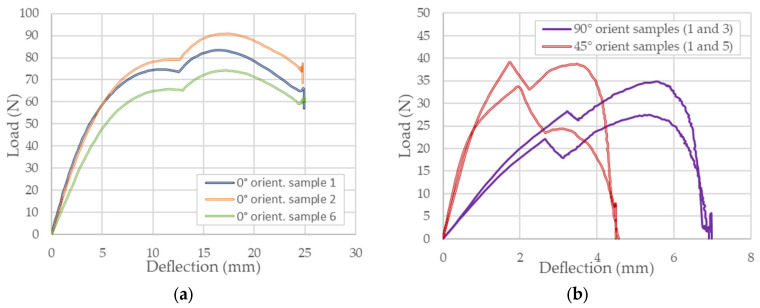
Sample plots of three-point bending load-deflection curves: (**a**) Flatwise specimens; (**b**) 45° flat-to-upright versus upright standing specimens.

Three typical samples of load-deflection curves recorded in the framework of three-point bending tests of flatwise printed PA12 SLS specimens are shown in Figure 3a, whereas several curves acquired for the 45° flat-to-upright and upright built specimens are presented in Figure 3b.

As may be seen, for the flatwise built oriented specimens, the maximum deflection exceeded 12 mm, which is considerably larger than the thickness of the specimen. On the contrary, the 45° flat-to-upright and the upright orientated specimens failed at much lower bending deflections of approximately 2 to 4 mm. Moreover, since large flexural deflections are sustained without fracture, a considerable shifting movement of flatwise printed specimens at the cylindrical supports was observed during each bending test.

In the figures above, the difference between the shapes of flexural load-deflection curves obtained for each specific build orientation (i.e., flatwise, 45° flat-to-upright, and upright) of PA12 SLS material under analysis may be distinguished. Thus, in the case of flatwise oriented specimens, the applied load is almost linearly proportional to the deflection only in the initial loading stage (see Figure 3a). Then the curve becomes nonlinear, and the load reaches its maximum in a plateau region, with an average maximum value of 80 N corresponding to a deflection of 10 to 12 mm. Although such a plateau signifies the occurrence of excessive plasticity, it finishes suddenly at a knee point that identifies the onset of specimen shifting upon the cylindrical supports of the three-point bending fixture. Next to the knee point, the load-deflection curve exhibits another steep trend increase followed by a leveling off and a slight decrease, indicating a new level of plasticity during the specimen shifting regime. After stopping the test, the specimen deflection recovered rapidly with about 60–70% from the maximum deformation.

A completely different response was exhibited by the specimens built with 45° flat-to-upright and upright orientation (see Figure 3b). That is, the 45° flat-to-upright specimens display a load-softening behavior with a primarily nonlinear elastic ascending branch up to the first flexural cracking load (equal to an average of 35 N reached at a deflection about half the specimen thickness). At this point, a sudden drop of load occurs (of 10% to 30%), followed by a slightly ascending post-elastic step region with all subsequent load values smaller than the first critical one. Then, a final descending trend until the final fracture (occurring at an average deflection value less than 5 mm) closes the curve. On the other hand, the upright specimens show a typical load-hardening behavior, with an initial nonlinear elastic ascending branch up to the first cracking load (equal to an average of 30 N at a deflection value which equals about the specimen thickness), identified through a slightly sudden drop of load (of 5% to 10%), followed in a first instance, by an ascending post-elastic region with a maximum load higher than the first critical one. Subsequently, it decreases smoothly until the final bending fracture (corresponding to an average deflection of 7 mm).

**Figure 4:**
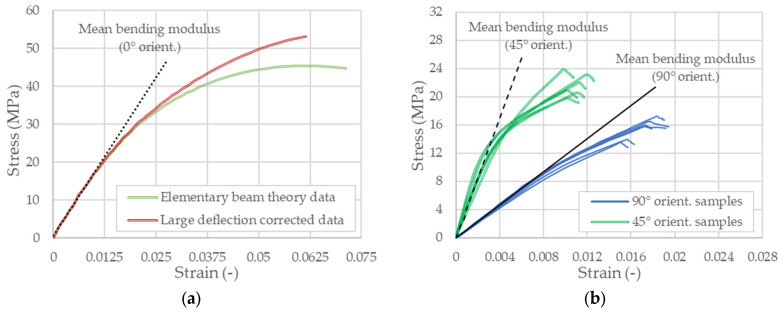
Plots of bending stress vs. strain: (**a**) Flatwise specimens; (**b**) 45° flat-to-upright and upright standing specimens.

Equation (2) was employed to obtain the strain from the deflection based on the elementary beam theory. However, the influence of large displacements on both the strain and bending stress was considered using Equations (3) and (4). Figure 4a shows the typical $\sigma_{\mathrm{eb}}-\varepsilon_{\mathrm{eb}}$ and $\sigma_{\mathrm{ld}}-\varepsilon_{\mathrm{ld}}$ curves obtained for a flatwise PA12 specimen. These curves are similar in the small strain region, while the discrepancy between the results becomes significant in the large strain region.

Figure 4b shows all the three-point bending stress-strain curves obtained for both 45° flat-to-upright and upright specimens. Since the strains are typically minor, no differences exist between the elementary beam theory results and those based on the large displacement formulation. However, a significant difference in the stiffness response can be observed, with a lower value but a minor deviation from linearity up to the fracture onset in the case of upright oriented specimens compared to the specimens built with 45° flat-to-upright orientation.

**Figure 5:**
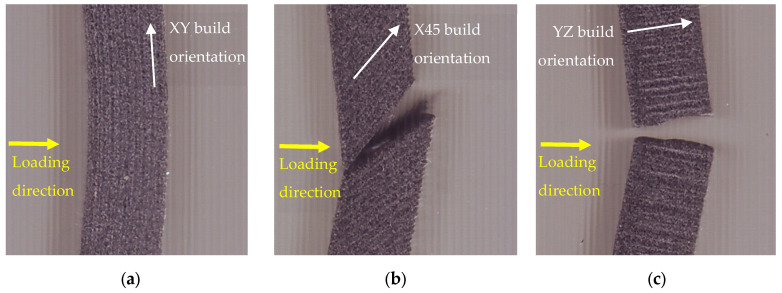
The characteristic failure modes of PA12 specimens under three-point bending tests (**a**) Flatwise orientation; (**b**) 45° flat-to-upright orientation; (**c**) upright orientation.

Directly related to the shape of load-deflection curves reported above, the characteristic failure modes for the build orientations of PA12 SLS in the adopted three-point bending test conditions are shown in Figure 5.

As for the three-point bending tests performed on flatwise build orientated specimens indicated in Figure 5a, it is worth noting that no fracture was observed in these specimens, even for excessive deflections. Such a non-breakability was not reported so far; nonetheless, some dissimilarities against the data reported in literature could result from different printing parameters. More remote, all the specimens having 45° flat-to-upright and upright building orientation underwent a much smaller deformation at failure, indicating a reduced ductility and a fracture plane that follows the layerwise microstructure of the two involved printing directions (see Figure 5b,c).

### 3.2. SEM Evaluation

**Figure 6:**
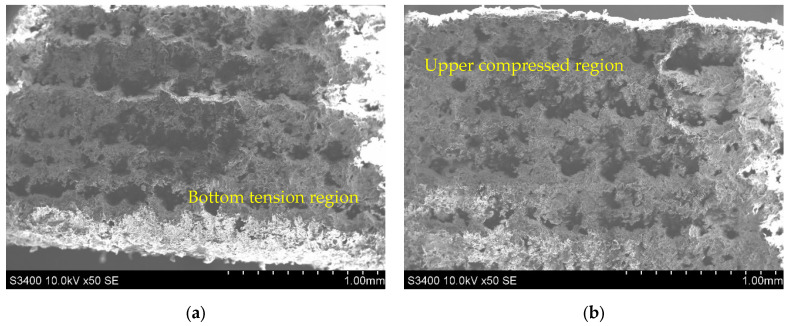
SEM image on the lateral side of a flatwise oriented specimen at the midspan length: (**a**) tension (bottom) area; (**b**) compressed (upper) area.

Scanning electron microscopy analysis was employed using an SEM Hitachi S-3400 N type-II (Hitachi, Tokyo, Japan) to determine the specific micro-mechanisms of failure and, particularly, the existence of printing flaws that could condition the three-point bending test results. Figure 6 shows the SEM images acquired from the lateral side of a flatwise oriented specimen in the permanent deformed state obtained after stopping the bending test. Due to the specimen retraction back when removing the applied load, only the state of permanent deformation was seized, as the elastic portion of the strain is being recovered.

As with static flexure loading, the flatwise oriented specimens under the three-point bending test show that the midspan region near the bottom tension area (see Figure 6a) has a different and relatively rougher surface compared to the upper compressed region (see Figure 6b). Moreover, there is evidence of increased delamination in the tension area instead of the compressed one that typically distorts like a plissé shade or accordion on the microscopic images.

**Figure 7:**
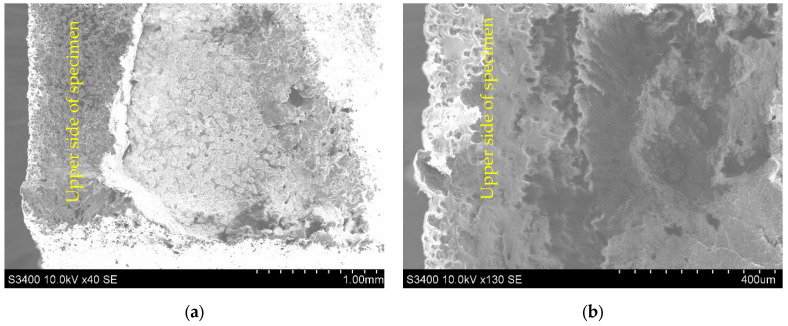
SEM image of fractured surfaces: (**a**) Flat-to-upright oriented specimen; (**b**) Upright standing oriented specimen.

The relatively smooth fracture surfaces of X45 flat-to-upright and upright standing specimens exhibit nearly similar fractographic features (see Figure 7a,b). In particular, in the case of X45 flat-to-upright specimens, a cleavage step fracture occurs, which indicates a composite underwent quasi-brittle fracture.

As shown in Figure 7b, at high resolution, it is confirmed that bending induces craze formation followed by tearing of the ligaments. On the other hand, the intergranular fracture between layers predominantly took place, and the granulated nature of PA12 SLS micro-structure is displayed, with the sizes of granules being similar with that of the original powder. These types of directionally-dependent fracture behavior under different loading conditions have also been observed in other PA12 AM materials processed via selective laser sintering [15,28,42,43].

### 3.3. Bending Strength and Flexural Modulus

The obtained results of bending strength and modulus of elasticity in flexure for the three particular build orientations of PA12 SLS are reported in Table 1. The following statistical descriptive parameters were calculated with SPSS software according to ESDU 91041b [44]: the mean value, the two-sided 95% confidence interval for the mean value, the standard deviation, and the coefficient of variation. One sample was excluded from the analysis as an extreme outlier for each build orientation in question.

The XY orientation (flatwise) showed the highest bending strength among the three tested build-orientations, while the lowest average bending strength value was obtained for the ZY orientation (upright standing). The highest flexural modulus was achieved by the 45X orientation (flat-to-upright). The ZY orientation (upright standing) had the lowest mean value of flexural modulus of elasticity. The highest statistical dispersion of bending strength, expressed by the coefficient of variation, was obtained for the ZY orientation (upright), while the highest dispersion of flexural modulus was obtained for the 45X orientation (flat-to-upright).

Kolmogorov-Smirnov and Shapiro-Wilk tests were performed to measure the probability that each build orientation sample of PA12 SLS comes from a population with normal distribution [45]. In this endeavor, the values of K–S and S–W statistics in line with their related significance levels (*p*) were determined to establish the compliance of test results with a normal distribution. The following statistical assumptions were considered: the level of significance (α=0.05), the null hypothesis H0: i.e., the population of the dependent variable from which the samples were extracted follows a normal distribution (for p>α), and the alternative hypothesis Ha: i.e., the population from which the sample was extracted does not follow a normal distribution (for p<α). Table 2 summarizes the results of the normality check performed in SPSS. It can be observed that both the Kolmogorov-Smirnov test and the Shapiro-Wilk test data suggest that bending strength and flexural modulus follow a normal distribution for each build orientation of PA12 SLS under analysis.

In the next stage, a one-way analysis of variance (ANOVA with one variable) was carried out to study the strength and elasticity statistics of PA12 SLS under three-point bending tests. In particular, Levene’s test was employed to analyze the homogeneity of variance within the three build orientations groups for each dependent variable. Virtually, the null hypothesis H0 assumes that the variances in the three orientation groups are homogeneous (*p* > α), while they are heterogeneous for the alternative hypothesis Ha, (*p* < α = 0.05). The results related to bending strength variable indicate homogeneous variances in the three orientation groups (*p* = 0.218 > α = 0.05), whereas the data calculated for the modulus of elasticity in flexure return the heterogeneity of variances (*p* = 0.003 < α = 0.05) between the groups (based on the mean and trimmed mean values). In addition, the ANOVA test results show that the level of probability between the building orientation groups for both dependent variables (*p* < α = 0.05) rejects the null hypothesis and supports the alternative one (i.e., at least two mean values differ from each other).

Although the ANOVA test shows the overall significance of three-point bending test results, it does not specify where the actual differences lie. Therefore, after running the ANOVA one-way analysis, Tukey’s HSD and Games-Howell tests have been considered to evaluate which mean value of the three build orientation groups (compared with each other) are different as it compares all possible pairs of means. Reasonable differences between the results of bending strength and flexural elasticity modulus were obtained for the assumed significance *p*-level of 0.05.

### 3.4. Reliability of Bending Strength

**Figure 8:**
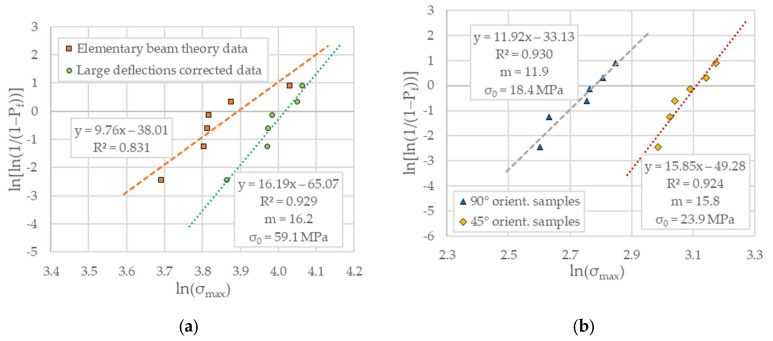
The Weibull parameters fitting plots: (**a**) Flatwise specimens; (**b**) 45° flat-to-upright and upright oriented specimens.

The Weibull moduli and the values of characteristic strengths obtained through the linear regression analysis are shown in Figure 8. The best-fit straight lines with the slightest errors measured on the ordinate axis return the data necessary to evaluate failure probabilities for each additively manufacturing build orientation in question.

In the above figure, one may notice that the experimental data related to flatwise oriented specimens, calculated by incorporating the effects of large displacements, exhibits the highest Weibull modulus of 16.2 with a correlation coefficient of 0.93. It appears that, for this orientation, the large deflection corrected data shows the lowest dispersion of bending strength. On the other side, the lowest Weibull modulus of 11.9 with a correlation coefficient of 0.93 was determined for the case of upright build orientation. However, the differences between the Weibull modulus of flexural strength for the three tested PA12 build orientations were not very large but pretty significant as the difference between the highest and the lowest value equals 4.3.

## 4. Discussion

The commercial datasheet of Nylon 12 material property data provided by the manufacturer of the presently used 3D printer machine specifies some reference values for the ultimate tensile strength, bending strength, and flexural elasticity modulus, regardless of a particular build orientation or powder refresh rate. Neither specific printing parameters nor details regarding the testing conditions are recommended to benchmark these testimonial values. However, it is apparent that the flexural strength performance of SLS printed Nylon 12 is superior to its tensile strength. In point of fact, this difference seems reasonable as it could be associated with internal flaws that are prone to occur within the additively manufactured parts or specimens. It is known that in the selective laser sintering process, small interface gaps often arise due to uneven heat distribution, lack of an adequate laser power supply, and/or inadequate temperature control. Besides, from the point of view of test specificity, while only half of the specimen experiences tension when undergoing bending loads, it carries out tension over the entire cross-section area in the case of standard tensile testing, which implies an increased probability of internal flaws, resulting in more likely failure initiation locations, and consequently, a lower value of tensile strength. In this regard, one should also note that compared to the standard tensile tests, which typically provide essential information about the mechanical behavior of metals, the bending tests are specifically more helpful in predicting the mechanical properties and failure modes of AM polymeric materials, especially for those designed to carry and transfer the out-of-plane loads. All the same, it turned out that most of the studies so far have focused on the tensile properties of AM PA12 SLS [19,20,21,23,24,43]. In particular, the three-point bending test seems to have significant advantages compared to the ordinary tensile test because it uses smaller coupons that are easier to handle, requires less time to test, lower testing machine capability, and not least, additional tabs are not needed to prevent the possible damages at the clamping grips [46]. On the other hand, it has the disadvantage that the applied load acts at the midspan of the specimen resulting in a spatially complex stress state, which is rather undesirable from the point of view of results interpretation.

Once the three-point bending static responses of Nylon 12 (PA12) laser sintered specimens were obtained experimentally, their benchmarking with the representative reference data from the scientific literature is rational. Thus, following a systematic and up-to-date literature review, the available bending properties corresponding to different printing parameters of PA12 SLS produced specimens are reported in Appendix A (Table A1). Based on the data presented in this table, some differences in bending strength and flexural elasticity modulus may be observed from one study to another. Nevertheless, a direct link between the values cannot be made due to the technological heterogeneity of the employed SLS platforms as well as different printing variables such as energy level, layer thickness, printing speed, and the volume ratio of reused to virgin powder. On top of that, due to the apparent lack of directionally dependent data in this review table, a need was felt to investigate the effect of building orientation on the flexural properties of PA12 specimens, additively manufactured using a benchtop industrial SLS platform (Formlabs Fuse 1), as it has not yet been studied so far. Hence, three-point bending tests were conducted to provide valuable insights into their strength and stiffness performance under the combined influence of tensile, compression, and shear stresses.

There is a significant build orientation-dependent anisotropy in bending strength and flexural elasticity modulus. The X45 flat-to-upright specimens consistently performed the best in terms of modulus of elasticity in flexure. In contrast, the flatwise-oriented specimens showed superior bending strength but a low modulus elasticity in flexure compared to X45 flat-to-upright specimens. The weakest values of both bending strength and modulus of elasticity in flexure were obtained for the upright standing oriented specimens. This behavior is the apparent consequence of the layered-like additively manufacturing approach as it comes with the so-called intra- and inter-layer powder packing capabilities along with the specific printing parameters of any selective laser sintering process [19,22,27,30,47,48]. That is, for relatively short scanning vector lengths, the subsequent adjoining 3D printed lines fuse firmly together within the same layer of sintered material and lead to an adequate intra-layer powder packing, as they do not have enough time to cool down before fusing a new line. As the length of the scanning vector increases, the time for cooling down may become large enough to generate an inappropriate intra-layer powder packing. After all, the time required to start processing a new layer is generally sufficient to allow for a certain degree of solidification in the previously completed underlayer, resulting in the formation of small interface gaps due to poor bond adhesion between the superimposed layers. These interface gaps between the subsequent sintered layers were also called “grooves” by Coy et al. [27], as they act as stress risers that create potential weak areas and, consequently, lead to inter-layer powder packing difficulties. In this regard, one may recognize that the directionally dependent anisotropy of elasticity modulus in flexure can be mainly attributed to intra-layer powder packing difficulties in the horizontal plane, while the bending strength dependent anisotropy is owing to the aversive effects of inter-layer powder packing in the vertical plane. Thus, by orienting the specimens at 45° simultaneously avoids both the weak orientation of the inter-layer interface against the main direction of normal stresses, as observed in the upright standing specimens and, the unfavorable influences of poor intra-layer powder packing in the horizontal plane, as it was particularly distinguished in the flatwise manufactured PA12 specimens. However, the mean value of bending strength for X45 flat-to-upright specimens is significantly lower than that obtained in the case of flatwise manufactured specimens.

Although both the load-deflection and stress-strain curves obtained under three-point bending loading conditions provide essential information about the mechanical behavior of additively manufactured PA12 SLS materials, previous publications omitted to present in detail these diagrams. Thus, it was shown that the general trend of bending stress-strain curves differs quite a lot from the actual tensile stress-strain curves reported in the literature. This observation confirms both the thesis of an accentuated anisotropy in bending compared to pure tensile loading as well as the fact that the directional dependence of bending properties is robustly imparted by the printing build orientation rather than a specific behavior of PA12 raw material. In this regard, for the case of flatwise oriented specimens, comparisons have shown that the elementary beam theory as per ISO 178 [36] gives significantly lower bending strength predicted values than those determined by the equation provided by ISO 14125 [37] owning to large deflection effects. Contrarily, the bending stress-strain curves of X45 flat-to-upright and upright oriented specimens are not sensitive to the effects of geometric nonlinearities. Nevertheless, the deflection does not influence the measurement of bending elasticity modulus.

One-way factorial analysis of variance (ANOVA) and post hoc tests were carried out to statistically analyze the bending strength and modulus of elasticity in flexure for the three build orientation groups of PA12 SLS specimens (i.e., 0° flatwise, 45° flat-to-upright and 90° upright standing, respectively). The normal distribution of variables was assessed through Kolmogorov-Smirnov and Shapiro-Wilk tests. All data were normally distributed and expressed as the mean plus/minus standard deviation, with a value of *p* less than 0.05 considered statistically significant. In addition, Tukey HSD was used to identify if one mean value differs significantly from another. Although the method is not statistically robust, being sensitive to the condition that the mean values must follow a normal distribution, many researchers claim it is meaningful since it avoids Type II errors (i.e., reject the equality when they are not different) [49]. In order to ensure that there is complete equality in the variances of the differences among the variations of related groups, Levene’s test was considered as is commonly utilized to examine the plausibility of homoscedasticity for data from at least three samples [50].

In general, the polymeric AM materials exhibit significant scattering of mechanical properties data, and therefore it is difficult to determine reliable bending strength values only by testing several independent specimens. In this respect, the presence of printing flaws in AM PA12 SLS specimens suggests the employment of a two-parameter Weibull equation, which allows predicting failure probability in terms of Weibull modulus and characteristic strength. While the former can be seen as an intrinsic material property associated with the width of data distribution and consequently describes the scatter of its bending strength, the latter is a scale parameter intended to measure the strengths of different orientations of AM PA12 SLS material, tested under similar conditions.

For the three particular PA12 SLS build orientations under discussion here, the calculated Weibull moduli and characteristic strengths are believed to have representative values under the reserve of unchanged printing parameters specified in Section 2.1. Indeed, it needs to keep in mind that the bending response of such materials may vary significantly depending on the process conditions, platform type, the source of PA12 powder, and not the last, the finishes of part surfaces.

**Figure 9:**
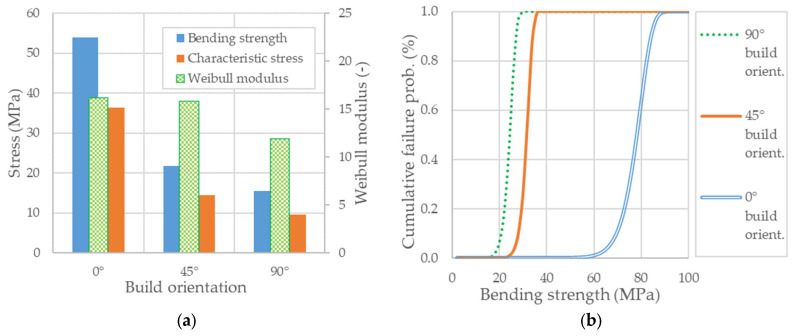
Reliability plots of bending strength: (**a**) Bending strength versus characteristic stress and Weibull modulus; (**b**) Cumulative failure probability curves.

The previous section showed that with the change of build orientation from 90° to 0°, the Weibull modulus appears to be on a growth trend (see also Figure 9a). Thus, a higher value indicates a lower strength dispersion, which means that the three-point bending fracture mechanism is transiting from ductile in the case of flatwise built orientation to quasi-brittle for upright standing oriented specimens. Such a response is desirable as the AM build orientations that exhibit large Weibull moduli are more predictable and less likely to break at much lower stress than the measured mean values. Moreover, as Figure 8 illustrates, according to decreasing strength, the parameters m (i.e., the Weibull modulus) and $\sigma_{0}$ (i.e., the characteristic strength) appear to be correlated, meaning that the lower bending strength exhibits less scatter, which shows a more consistent—or a more uniform—flaw population. For all that, the validity of the Weibull approach can be proved by verifying its ability to scale the strength values obtained for a particular testing configuration to another one or a different specimen size. It is beyond the scope of this work to address this issue, but its implications in terms of probabilities of failure for the case of AM parts made of PA12 SLS are worth being further explored. Despite this, a straightforward application is best illustrated in Figure 9b, which displays the cumulative failure probability distribution as a function of the characteristic stress according to the analytical expression (A6).

As the characteristic strength in Figure 9a indicates the location of the cumulative distribution function relative to the abscissa axis one may expect its movement along, depending on the specimen size or testing procedure, due to a relevant change in the highly stressed volume of material. Since the structural design methodologies for AM parts made of PA12 SLS materials may require specific information relative to a certain level of failure probability as a function of the characteristic stress, such a procedure based on Weibull statistics could provide an appropriate and valuable solution.

## 5. Conclusions

There is a significant build orientation-dependent anisotropy in bending strength and flexural elasticity modulus. The X45 flat-to-upright specimens consistently performed the best in terms of modulus of elasticity in flexure. Contrarily, the flatwise-oriented specimens showed superior bending strength but a low modulus elasticity in flexure relative to X45 flat-to-upright specimens. The weakest values of both bending strength and modulus of elasticity in flexure were obtained for the upright standing oriented specimens.

The directionally dependent anisotropy of elasticity modulus in flexure can be attributed mainly to intra-layer powder packing difficulties in the horizontal plane. Quite the reverse, the bending strength-dependent anisotropy appears to be directly linked to the aversive effects of inter-layer powder packing in the vertical plane.

The general trend of bending stress-strain curves relative to the build orientation differs quite a lot from the actual tensile stress-strain curves reported in the literature. This observation confirms both the thesis of an accentuated anisotropy in bending compared to pure tensile loading as well as the fact that the directional dependence of bending properties is robustly imparted by the build orientation rather than a specific behavior of PA12 raw material.

In the case of flatwise oriented specimens, comparisons have shown that the elementary beam theory gives significantly lower bending strength predicted values than those considering the effect of large deflections.

Nylon 12 SLS exhibit significant scattering of mechanical properties data, and therefore it is difficult to determine reliable bending strength values only by testing several independent specimens. In this respect, the presence of printing flaws in AM specimens made of PA12 SLS suggests the employment of a two-parameter Weibull equation, which allows predicting failure probability in terms of Weibull modulus and characteristic strength.

## Figures and Tables

**Table 1:** Descriptive statistics data for three built orientations of PA12 SLS under the three-point bending tests.

Build Orientation	Descriptive Statistics		Dependent Variable (MPa)	
			Bending Strength	Flexural Elasticity Modulus
Flatwise (0°)	Mean value		53.9	1541.9
	95% Conf. interval for Mean	Lower Bound	49.9	1316.2
		Upper Bound	57.8	1767.7
	Std. Deviation		3.76	215.1
	Coefficient of variation		0.069	0.139
Flat-to-upright (45°)	Mean value		21.72	4577.2
	95% Conf. interval for Mean	Lower Bound	20.06	3719.1
		Upper Bound	23.38	5435.4
	Std. Deviation		1.58	817.69
	Coefficient of variation		0.072	0.178
Upright (90°)	Mean value		15.46	1135.3
	95% Conf. interval for Mean	Lower Bound	13.92	1079.3
		Upper Bound	17.00	1191.3
	Std. Deviation		1.47	53.39
	Coefficient of variation		0.095	0.047

**Table 2:** Results of Kolmogorov-Smirnov and Shapiro-Wilk tests for normality check of bending strength and flexural modulus.

Dependent Variable (MPa)	Build Orientation	Kolmogorov-Smirnov *		Shapiro-Wilk	
		Statistics	*p* (Sig.)	Statistics	*p* (Sig.)
Bending strength	Flatwise (0°)	0.249	0.200	0.912	0.450
	Flat-to-upright (45°)	0.194	0.200	0.954	0.773
	Upright (90°)	0.238	0.200	0.920	0.504
Flexural modulus	Flatwise (0°)	0.347	0.070 **	0.748	0.298 **
	Flat-to-upright (45°)	0.297	0.106	0.912	0.450
	Upright (90°)	0.146	0.200	0.975	0.924

* Lilliefors Significance Correction is considered. ** The value represents the lower bound of the true significance.

## Data Availability

The data presented in this study are available in the article.

## Appendix A

**Table A1:** Summary of representative mean values of static flexural properties for PA12 SLS, collected from the scientific literature.

Ref.	SLS Platform Type	Print Orient. (deg)	Bending Strength (MPa)	Flexural Modulus (MPa)	Laser Power (W)	Layer Thick. (mm)	Testing Specs *	Powder Type and Supplier
[22]	3D Systems Vanguard SI **	0°	60.3	1104	11	0.1	ISO 178 [32]	Duraform PA (3D Systems)
		45°	-	-				
		90°	57.7	1054				
[27]	EOS Formiga P100 ***	0°	-	2077 *	-	-	ISO 178 [32]	PA 2200 (EOS)
		45°	-	2088 *				
		90°	-	2088 *				
[28]	- ***	0°	86	546	3.8	0.2	-	Duraform PA (3D Systems)
		45°	-	-				
		90°	-	-				
[29]	DTM Sinterstation 2000 System ***	0°	73.5	1666	6	0.1	ISO 178 [32]	PA 2200 (EOS)
		45°	-	-				
		90°	-	-				
[30]	DTM Sinterstation 2000 System ***	0°	46.7	1710	7	0.1	ISO 178 [32]	PA 2200 (EOS)
		45°	-	-				
		90°	-	-				
[31]	EOS Formiga P100 ***	0°	59.2	551	25	-	ASTM D 5045-99	PA 2200 (EOS)
		45°	46.3	433				
		90°	19.9	345				
[32]	3D Systems Vanguard SI ****	0°	66	1166	2.8	0.1	ASTM D 790	Duraform PA (3D Systems)
		45°	-	-				
		90°	70	1281				

* The values were calculated based on the empirical relationships provided within the paper [27] and correspond to a specimen thickness of 4 mm. ** Only the virgin powder was employed in the study. *** No powder mixture ratio (fresh/reused) specifications were available. **** The powder was artificially aged to achieve uniform characteristics over different printing sessions.

## Appendix B

With the provision that for the particular case of three-point bending, only half of the specimen of volume V effectively undergoes tension [39,40], as the normal stress distribution is varying linearly across the thickness of the specimen, from a maximum value on the tensioned face ($\sigma_{\max}$), to a minimum value on the opposite compressed face ($\sigma_{\min}=-\sigma_{\max}$), one may write:

(A1) $${\sigma (y)=\sigma }_{\max}\left(1-\frac{2y}{h}\right).$$

where h denotes the thickness of the specimen and y is the ordinate axis of a local coordinate system attached to the lower face of its rectangular cross-section. By integrating the Equation (7) over the tensioned volume V, defined by 0 ≤ y ≤ h/2, yields to:

(A2) $${1\;-\;P}_{f}(\sigma )=\exp\left[-{\left(\frac{\sigma_{\max}}{\sigma_{0}}\right)}^{m}\frac{V}{2{\left(m+1\right)}^{2}}\right],$$

where the stress $\sigma_{\max}$ which causes the probability of material failure to be 50%, is sometimes termed as the modulus of rupture (i.e., the strength index determined through the bending test). Then, the above equation was linearized by natural log transformation, which enabled to fit the Weibull parameters from the experimental data:

(A3) $$\ln\left[\ln\left(\frac{1}{1-P_{f}(\sigma )\;}\right)\right]=m\cdot {\ln(\sigma }_{\max})+\ln\left[\frac{V}{2{\left(m+1\right)}^{2}\cdot {\;\sigma }_{0}{}^{m}}\right].$$

The probability of failure was estimated using the equation [41]:

(A4) $$P_{f}=\frac{\left(i-0.5\right)}{n},$$

where n returns the total number of tested specimens for a given build orientation, and i is the position of the test specimen after they were ranked by increasing the magnitude of bending strength. The standard calculation procedure is to regress linearly the ${\ln[\ln(1\;-\;P}_{f}\left(\sigma ))\right]$ data against the values of ln(bending strength). Thus, by plotting the left-hand side of Equation (A3) against ${\ln(\sigma }_{\max})$, would lead to a straight-line fit of data, with the slope m and the intercept ln[V/$2{\left(m+1\right)}^{2}\cdot {\;\sigma }_{0}{}^{m}$].

As long as the characteristic strength (sometimes termed as the scale factor) is the value of stress for which the probability of failure equals 63.2%, that is the left side in the Equation (A3) vanishes (i.e., ln[ln(${1\;-\;P}_{f}\left(\sigma \right)$)] = 0), one may write:

(A5) $$\ln\left[\frac{V}{2{\left(m+1\right)}^{2}\cdot {\;\sigma }_{0}{}^{m}}\right]=-m\cdot {\ln(\sigma }_{\max}),$$

which, after some simple mathematical manipulations, leads to the following expression of the characteristic stress under the three-point bending loading condition:

(A6) $$\sigma_{0}={\left\{\frac{V}{2{\left(m+1\right)}^{2}\cdot \exp\left[-m\cdot {\ln(\sigma }_{\max})\right]}\right\}}^{\frac{1}{m}}.$$

where V is the effective volume of the specimen experiencing tension stresses.
